# Complete Genome Sequence of *Enterococcus faecalis* Strain W11 Isolated from an Algal Food Product

**DOI:** 10.1128/genomeA.01037-16

**Published:** 2016-09-29

**Authors:** Yuki Doi, Noboru Takizawa

**Affiliations:** Department of Applied Chemistry and Biotechnology, Faculty of Engineering, Okayama University of Science, Okayama, Okayama, Japan

## Abstract

Here, we report the complete genome sequence of *Enterococcus faecalis* strain W11 isolated from an algal food product in Japan. This study should facilitate the identification of a novel mechanism of glycerol metabolic control in lactic acid bacteria.

## GENOME ANNOUNCEMENT

*Enterococcus faecalis* is one of the lactic acid bacteria in the human intestinal tract that can use glycerol as a carbon source (1). *E. faecalis* strain W11, isolated from salt-preserved seaweed (wakame; *Undaria pinnatifida*), consumes glycerol more efficiently than other strains, with or without oxygen (2). In particular, although previous studies reported that *E. faecalis* strains require exogenous fumarate for the oxidation of NADH generated from anaerobic glycerol metabolism (3, 4), W11 can metabolize glycerol without exogenous fumarate in the absence of oxygen (2).

Genomic DNA of *E. faecalis* strain W11 was isolated using DNeasy blood and tissue kits (Qiagen, Hilden, Germany), and 1 µg of genomic DNA was fragmented to 350 bp using the Covaris S220 (Covaris, Woburn, MA, USA). The fragmented DNA was end-repaired, A-tailed, and ligated to paired-end adapters using the TruSeq DNA sample preparation version 2 kit (Illumina, San Diego, CA, USA). The DNA fragments containing adapter sequences were separated on a 2% agarose gel and then the 450- to 550-bp band collected using the QIAquick gel extraction kit (Qiagen) was enhanced by PCR using adapter-specific primers. After cluster generation with the Illumina cBot cluster-generation system using the TruSeq PE cluster kit version 3-cBot-HS (Illumina), paired-end sequencing was performed on the Illumina HiSeq 2000 (Illumina) using a TruSeq SBS kit version 3-HS (Illumina). Raw reads were trimmed and *de novo* assembled using Edena version 3 (5). Gaps between contigs were closed by PCR followed by Sanger sequencing, and the resultant complete genome sequence was annotated using RAST (6).

The circular genome of W11 (2,704,865 bp) contained 2,537 coding sequences, 12 rRNA sequences, and 51 tRNA sequences. The average GC content was 37.7%. Notably, sequences around EFW11_0067 (75,432 to 76,085) and EFW11_0082 (84,062 to 85,297) were significantly different from those of other *E. faecalis* strains, such as V583 and JH2-2. EFW11_0067 is a homolog of the EF0072 gene encoding the transcriptional regulator Ers for glycerol metabolism, and EFW11_0082 is a homolog of the EF0082 gene encoding a putative glycerol uptake protein regulated by Ers (7, 8). These findings show the possibility that strain W11 has a unique mechanism of metabolic control for fumarate-independent glycerol utilization. The complete genome sequence of W11 should facilitate the investigation of this mechanism.

### Accession number(s).

The genome sequence of *E. faecalis* strain W11 has been deposited in DDBJ/EMBL/GenBank under the accession number AP017623.
